# Genomic and bioinformatics analysis of human adenovirus type 37: New insights into corneal tropism

**DOI:** 10.1186/1471-2164-9-213

**Published:** 2008-05-09

**Authors:** Christopher M Robinson, Fatemeh Shariati, Allison F Gillaspy, David W Dyer, James Chodosh

**Affiliations:** 1 Molecular Pathogenesis of Eye Infection Research Center, Dean A. McGee Eye Institute, 608 Stanton L. Young Blvd., Oklahoma City, OK 73104, USA; 2 Department of Ophthalmology, University of Oklahoma Health Sciences Center, 1100 North Lindsay, Oklahoma City, OK 73104, USA; 3 Department of Microbiology & Immunology, University of Oklahoma Health Sciences Center, 1100 North Lindsay, Oklahoma City, OK 73104, USA; 4 Department of Cell Biology, University of Oklahoma Health Sciences Center, 1100 North Lindsay, Oklahoma City, OK 73104, USA; 5 Laboratory for Genomics and Bioinformatics, University of Oklahoma Health Sciences Center, 1100 North Lindsay, Oklahoma City, OK 73104, USA

## Abstract

**Background:**

Human adenovirus type 37 (HAdV-37) is a major etiologic agent of epidemic keratoconjunctivitis, a common and severe eye infection associated with long-term visual morbidity due to persistent corneal inflammation. While HAdV-37 has been known for over 20 years as an important cause, the complete genome sequence of this serotype has yet to be reported. A detailed bioinformatics analysis of the genome sequence of HAdV-37 is extremely important to understanding its unique pathogenicity in the eye.

**Results:**

We sequenced and annotated the complete genome of HAdV-37, and performed genomic and bioinformatics comparisons with other HAdVs to identify differences that might underlie the unique corneal tropism of HAdV-37. Global pairwise genome alignment with HAdV-9, a human species D adenovirus not associated with corneal infection, revealed areas of non-conserved sequence principally in genes for the virus fiber (site of host cell binding), penton (host cell internalization signal), hexon (principal viral capsid structural protein), and E3 (site of several genes that mediate evasion of the host immune system). Phylogenetic analysis revealed close similarities between predicted proteins from HAdV-37 of species D and HAdVs from species B and E. However, virtual 2D gel analyses of predicted viral proteins uncovered unexpected differences in pI and/or size of specific proteins thought to be highly similar by phylogenetics.

**Conclusion:**

This genomic and bioinformatics analysis of the HAdV-37 genome provides a valuable tool for understanding the corneal tropism of this clinically important virus. Although disparities between HAdV-37 and other HAdV within species D in genes encoding structural and host receptor-binding proteins were to some extent expected, differences in the E3 region suggest as yet unknown roles for this area of the genome. The whole genome comparisons and virtual 2D gel analyses reported herein suggest potent areas for future studies.

## Background

Adenoviruses (AdV) in the Adenoviridae family have been divided into four genera: *Mastadenovirus, Aviadenovirus, Atadenovirus*, and *Siadenovirus *[1]. The AdV was first isolated from human adenoids and characterized by two different research teams [2,3]. Human AdV (HAdV) fall within the genus *Mastadenovirus*, and cause a wide array of diseases including acute respiratory disease, gastroenteritis, and ocular surface infection [4-6]. The AdV is non-enveloped with a double stranded linear genome that ranges from 26 to 45 kb in size. The icosahedral shaped capsid ranges from 70 to 100 nanometers in diameter [7]. There are 51 known HAdV serotypes classified into 6 species (A-F), based on restriction enzyme analysis and hemaglutination assays, later confirmed by genome analyses and phylogenetic calculations. Recently, a proposed fifty-second HAdV serotype was identified and placed into a new species G [8].

HAdV-37 was originally isolated in 1976 from 62 eyes and 9 genitourinary sites, and subsequently characterized as a new serotype in 1981 [9]. HAdV-37 is a major etiologic agent of epidemic keratoconjunctivitis, an explosive and highly contagious infection of the conjunctiva and cornea, and continues to cause outbreaks [10]. HAdV-37 also was recently implicated in the pathogenesis of obesity [11].

Although HAdV species D contains the most serotypes, complete sequence is available for only 6 – HAdV-9, HAdV-17, HAdV-26, HAdV-46, HAdV-48, and HAdV-49 – and none of these have been associated with epidemic keratoconjunctivitis. In this study, we have sequenced the complete genome of HAdV-37 and describe its overall organization. The HAdV-37 genome appears in most respects typical of other HAdV. However, global pairwise genome alignments, phylogenetic analyses, and *in silico *comparisons of putative viral proteins revealed unique characteristics of the genome, including areas of non-conserved sequence in the penton, hexon, E3, and fiber regions, and differences in size and/or pI of select predicted HAdV-37 proteins. Understanding the disparities between the HAdV-37 genome and those of species D HAdV with dissimilar tissue tropisms may lead to improved understanding of the genomic determinants of infection.

## Results

### General features

**Figure 1:**
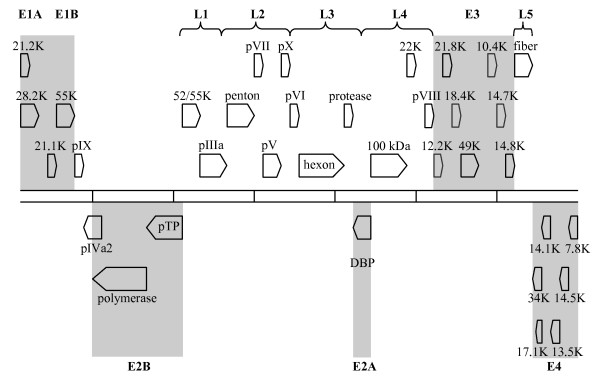
**Transcriptional map and genome organization of HAdV-37.** The two horizontal lines define the length of the HAdV-37 genome with each vertical line within them representing 5000 bps. The block arrows represent the predicted protein coding regions. The early transcription units (E1-E4) are highlighted. The late transcription units (L1-L5) are designated by parentheses.

The genome length of HAdV-37 was found to be 35,213 base pairs with a base composition of 22.8% A, 20.6% T, 28.3% C, 28.3% G. The 56.6% GC content is on the lower end of the 57–59% range previously reported for HAdVs within species D [7]. CpG dinucleotide analysis of HAdV-37 performed using FUZZNUC software [12] revealed 2389 CpG dinucleotides located within the genome (data not shown). We identified the predicted 4 early, 2 intermediate, and 5 late transcription regions similar to those described in other completely sequenced HAdVs (Figure 1), including 35 predicted coding sequences within the HAdV-37 genome and 8 hypothetical ORFs.

The 5' and 3' termini of the HAdV genome are composed of inverted terminal repeat (ITR) sequences which for HAdV-37 were determined to be 159 bp in length. These sites serve as replication origins for the virus [7]. The motif located at the extreme termini of the HAdV-37 genome consists of a CATCATCATAAT, which is unique among previously sequenced HAdV serotypes. Unique sequences for the extreme termini have been also observed in other HAdVs including HAdV-4 [13]. The conserved ATAATATACC motif within the ITR, which interacts with the terminal protein precursor (pTP) and polymerase complex during DNA replication [14], was determined at base pairs 8–17. A NFIII/Oct-1 recognition site (TATGCAAAT) was identified within the ITR of HAdV-37 at nucleotides 40–48. A Sp1 binding site (GGGGCGGA) was identified at nucleotides 73–80. Also, a NFI/CTFI (TGGGGCGGAGCCA) site was located at overlapping nucleotides 72–84.

### Global pairwise alignment

**Figure 2:**
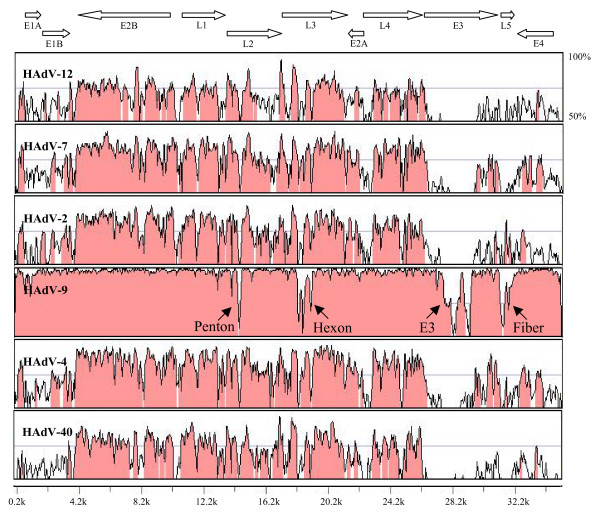
**Global pairwise sequence comparison of HAdV-37 with select serotypes from each of the 6 HAdV species (from top to bottom: species A to F) using the online sequence alignment program, mVISTA LAGAN.** Percent sequence conservation is reflected in the height of each data point along the y axis. The penton, hexon, E3, and fiber regions of HAdV-37 diverged from HAdV-9, another species D virus.

The mVISTA Limited Area Global Alignment of Nucleotides (LAGAN) tool was used to align and compare paired viral sequences [15]. We compared genomic sequence correspondence across the whole genome of HAdV-37 to representative HAdV serotypes from each of the six HAdV species. Comparison of the HAdV-37 genome with that of HAdV-9, also within species D, showed a much higher degree of conservation than with representative HAdVs from other species, but demonstrated disparity in the penton, hexon, E3, and fiber regions (Figure 2).

### Early genes

E1A is the first transcriptional unit to be expressed during infection [7]. A common RNA from this region is the source of several alternatively spliced E1A transcripts [16]. The E1A proteins regulate the transcription of viral and cellular genes [17,18]. Based on splice donor and acceptor sites, two putative proteins of 253 and 191 amino acids with corresponding molecular weights of 28.2 kDa and 21.2 kDa, respectively, were identified in the HAdV-37 genome (Table 1). The HAdV-37 E1A 21.2 kDa protein is 89% identical and 96% similar to the HAdV-9 homologue (Table 2). A protein corresponding to the previously predicted 10S protein from previous studies of HAdVs was not identified in our analysis. The predicted TATA box was identified at nucleotide 477 and the polyadenylation signal predicted to be at position 1451.

**Table 1:** Genome Organization of HAdV-37

Region	Gene Product	TATA box	ATG	Stop	Protein Length (aa)	Poly(A) signal
E1A	21.2 kDa	477	569–932*	1214–1425*	191	1451
	28.2 kDa	477	569–1118*	1214–1425*	253	1451
E1B	21.1 kDa	1525	1577	2125	182	3863
	55.2 kDa	1525	1882	3369	495	3863
Intermediate	IX	ND	3454	3858	134	3863
	hypothetical 20.5 kDa	ND	5765 c	5199 *c*	188	ND
	IVa2	ND	5514–5526*c	3902–5235*c	448	ND
	hypothetical 34.76 kDa	ND	6514	7503	329	ND
	hypothetical 16.57 kDa	ND	7786	8253	155	ND
E2A	DNA binding protein	ND	22777 *c*	21305 *c*	490	ND
E2B	pTP	ND	13495–13503*c	8325–10223*c	635	ND
	hypothetical 19.29 kDa	ND	8030 *c*	8563 c	177	ND
	DNA Polymerase	ND	8280 *c*	5005 *c*	1091	ND
	hypothetical 11.4 kDa	ND	9648	9983	111	ND
L1	52/55K	5827	10638	11762	374	13484
	hypothetical 8.58 kDa	ND	11781 *c*	12035 *c*	84	ND
	pIIIa	5827	11785	13476	563	13484
L2	Penton	5827	13530	15089	519	16982
	pVII	5827	15093	15683	196	16982
	V	5827	15716	16720	334	16982
	pX	5827	16750	16974	74	16982
L3	pVI	5827	17030	17734	234	21256
	Hexon	5827	17775	20642	949	21256
	Protease	5827	20627	21250	207	21256
L4	100K	5827	22794	24992	732	26507
	hypothetical 9.29 kDa	ND	24181 *c*	23936 *c*	81	ND
	22K	5827	24775	25188	137	26507
	pVIII	5827	25514	26197	227	26507
E3	12.2 kDa	25879	26198	26518	106	30837
	21.8 kDa	25879	26472	27062	196	30837
	18.6 kDa	25879	27029	27529	166	30837
	48.9 kDa	25879	27554	28840	428	30837
	hypothetical 31.6 kDa	25879	28867	29709	280	30837
	10.4 kDa	25879	29716	29991	91	30837
	14.72 kDa	25879	29994	30386	130	30837
	14.84 kDa	25879	30379	30771	130	30837
	U-exon	ND	31022 *c*	30873 *c*	49	ND
L5	Fiber	5827	31038	32135	365	32143
E4	7.8 kDa	34665	34564 *c*	34367 *c*	65	32184
	14.5 kDa	34665	34326 *c*	33934 *c*	130	32184
	13.56 kDa	34665	33937 *c*	33584 *c*	117	32184
	14.16 kDa	34665	33581 *c*	33219 *c*	120	32184
	17.13 kDa	34665	33122 *c*	32667 *c*	151	32184
	34.1 kDa	34665	33289 *c*	32411 *c*	292	32184

*Note: c*, complementary strand

*, predicted splice sites

**Table 2:** Percent identities/similarities of select HAdV-37 proteins and their homologs

Types/Species	E1A 21.2-kDa	E1B 21.1-kDa	IX	DNA binding protein	DNA polymerase	L1 52/55K	Penton
HAdV-12/A	41/65	46/77	56/79	52/78	78/91	78/92	74/86
HAdV-7/B	41/61	53/81	63/85	61/82	82/93	79/88	75/89
HAdV-2/C	35/46	50/78	52/83	57/76	83/93	75/88	68/82
HAdV-9/D	89/96	97/100	99/100	98/99	99/99	99/100	90/95
HAdV-4/E	42/63	50/79	62/86	60/79	83/93	77/87	77/88
HAdV-40/F	35/56	43/72	55/81	51/75	74/90	81/91	73/86

Types/Species	Hexon	L3 Protease	L4 100K	L4 pVIII	E3 14.7-kDa	Fiber	E4 14.5-kDa

HAdV-12/A	80/90	79/93	66/83	75/88	35/63	32/66	35/66
HAdV-7/B	85/93	84/93	69/84	82/93	47/68	32/57	42/78
HAdV-2/C	78/90	77/94	69/83	78/91	35/64	36/65	32/71
HAdV-9/D	90/95	100/100	98/99	99/100	98/98	76/89	100/100
HAdV-4/E	85/93	82/93	71/87	84/94	38/62	37/62	40/75
HAdV-40/F	80/92	78/94	66/84	80/90	30/53	31/59	37/66

Percent identities and similarities were calculated using FASTA3 (EMBL-EBI).

E1B proteins potentiate viral replication by blocking apoptosis. E1B 19K blocks the mitochondrial apoptosis pathway by inactivating BAK and BAX [19]. E1B 55K inhibits the ability of p53, the host tumor suppressor protein, to initiate cell cycle arrest [20,21]. The putative TATA box for the E1B messages was predicted at nucleotide 1525. Two predicted proteins of molecular weights of 21.1 and 55.2 kDa were identified within E1B which correspond to 19- and 55-kDa proteins, respectively, as reported for HAdV-9. Amino acid sequence analysis revealed that the predicted 21.1 kDa protein was 99% identical and 100% similar to the 19 kDa homologue found in the HAdV-9 genome (Table 2). The polyadenylation signal for these transcripts was predicted at nucleotide 3863.

The E2 region of the genome consists of two transcription units, E2A and E2B, which encode three proteins that are required for viral replication [7]. These three proteins are known as the DNA binding protein (DBP), terminal protein precursor (pTP), and DNA polymerase. The E2A 54.9 kDa DNA binding protein was identified on the complementary strand between nucleotides 21305 and 22777. Also on the complementary strand, but located within the E2B region, we identified the pTP and DNA polymerase. The polyadenylation signal for these transcripts was not identified.

The HAdV E3 region encodes proteins that modulate the host immune response to infection but are not required for viral growth *in vitro *[22,23]. HAdVs within species D have previously been suggested to encode eight ORFs within the E3 region [24,25]. Seven classical and one hypothetical E3 ORFs were identified in our annotation of HAdV-37. The predicted molecular weights for these are 12.2, 21.8, 18.6, 48.9, 31.6, 10.47, 14.7, and 14.8 kDa. The TATA box was predicted at nucleotide 25879 with a TATAAA motif. One polyadenylation signal for this transcription unit was identified at nucleotide 30837.

Open reading frames located in the E4 transcription unit produce proteins that have a wide variety of functions [26]. For example, E4 ORF 3 and E4 ORF 6 enhance the stability of late viral mRNAs and increase their export from the nucleus thereby increasing viral mRNA accumulation in the cytoplasm [26]. E4 ORF 6 also binds to p53 and can block apoptosis [27,28]. We found 6 predicted ORFs in HAdV-37 located on the complementary strand. Surprisingly, the E4 ORF 1 from the HAdV-37 genome was predicted at 65 amino acids in length corresponding to a molecular weight of 7.4 kDa. In contrast, the HAdV-9 homologue of E4 ORF 1 is 125 amino acids in length, and contains three regions essential for tumor transformation (region I, residues 34 to 41; region II, residues 89 to 91; region III 122 to 125). The E4 ORF 1 of HAdV-9 has structural similarity to other viral dUTPase enzymes [29,30]. ClustalW analysis of the HAdV-37 E4 ORF 1 compared to the HAdV-9 homologue revealed a 100% similarity from residues 61–125, including regions II and III and a truncated dUTPase domain. Further work will be needed to evaluate the significance of this truncation. The TATA box for this region was identified at nucleotide 34665 and the polyadenylation signal at nucleotide 32184.

### Intermediate genes

The intermediate genes of HAdV are IVa2 and IX. The IVa2 protein interacts with L1 52/55K during viral DNA packaging, and assists in the activation of the major late promoter (MLP) [14,31-33]. The HAdV-37 IVa2 gene, found on the complementary strand, was predicted using the splice site finder [34], with a 448 amino acid protein and 99% amino acid homology to HAdV-9 IVa2. The IX protein is a minor capsid protein and also assists in the activation of the major late promoter [35,36]. A coding sequence for a 13.7 kDa protein corresponding to IX was found at nucleotides 3454–3858.

### Late genes

The late transcription units of HAdVs are transcribed from the MLP, which consists of an inverted CAAT box (5777–5780 bp) and TATA box (5827–5832 bp). The late mRNAs have been grouped into five families (L1 to L5), based on the location of the polyadenylation signal. Proteins expressed by these five families are involved in capsid production for mature virions [7]. The L1 transcription unit encodes two proteins, 52/55K and IIIa. The 52/55K protein is involved in scaffolding of the capsid and therefore facilitates virus assembly [37]. The 52/55K protein also interacts with the intermediate gene product IVa2 to facilitate DNA packaging [31,33,38]. Polypeptide IIIa is a structural protein that has been located on the inner capsid surface below the penton base [39]. The 52/55K and polypeptide IIIa in HAdV-37 were predicted to have molecular weights of 42.2 kDa and 26.6 kDa, respectively. The predicted polyadenylation signal for the L1 region was found at nucleotide 13484.

The proteins encoded on the L2 transcription unit also are involved in capsid formation [7]. The penton base (protein III) is found at each of the 12 vertices of the virion [7]. The penton base contains an Arg-Gly-Asp (RGD) sequence which interacts with host integrins to induce internalization of the virus [40]. The HAdV-37 penton base is located at nucleotides 13530–15089. The length of the protein was predicted to be 519 amino acids with an estimated molecular weight of 58.4 kDa. The RGD sequence was located at amino acid position 309–311. The predicted protein was 100% identical to the previously published penton base protein identified for HAdV-37 [41]. The HAdV-37 penton base homologue is 90% identical and 95% similar to the predicted HAdV-9 penton base protein (Table 2). The V, VII, and X proteins constitute the HAdV core proteins and facilitate packaging of viral DNA within the capsid [42]. The HAdV-37 pVII protein-coding sequence was identified at nucleotides 15093–15683, and the protein was predicted to have a molecular weight of 21.7 kDa. Proteins with an amino acid length of 334 and 74 were predicted in the HAdV-37 genome for proteins V and X at nucleotides 15716–16720 and 16750–16974, respectively. The L2 transcripts share a putative polyadenylation signal at nucleotide 16982.

Three open reading frames corresponding to pVI, hexon, and protease proteins, with respective molecular weights of 25.5, 106.8, and 23.4 kDa, were identified within the L3 transcription unit. The pVI protein contains two nuclear export signals and two nuclear localization signals, and plays a role in transporting the hexon protein to the nucleus for viral assembly [43]. The C terminus of this protein has also been implicated in regulation of the viral protease [44]. The HAdV-37 pVI protein was located at nucleotides 17030–17734. This predicted 234 amino acid protein was 100% identical to its HAdV-17 homologue. The hexon protein, the most abundant virion component, constitutes 240 of the 252 subunits of the protein shell of the virus [7]. The HAdV-37 hexon protein is 949 amino acids in length and was nearly identical to that predicted by Ebner et al. [45]. Our sequence data suggests an additional 10 amino acids on the N-terminus of the protein, similar to the predicted hexon gene for HAdV-46 and HAdV-9, both within species D. The final protein encoded in the L3 transcription unit is the 23 kDa viral protease protein. The HAdV-37 homologue was predicted to be 207 amino acids in length. This protein cleaves other viral proteins allowing for assembly and viral maturation [46], and the transcript shares a predicted polyadenylation signal at nucleotide 21256 with other L3 transcripts.

Three L4 proteins, 100 kDa, pVIII, and 22 kDa, were predicted from our annotation. The 100 kDa protein is a nonstructural protein that assists in the translation of late viral mRNAs, and inhibits translation of cellular mRNAs [47,48]. More recently this protein has been implicated as a scaffold for trimerization of the hexon [49]. The predicted 100 kDa protein for HAdV-37 genome was 732 amino acids in length and had a molecular weight of 82.3 kDa. This protein is 98% identical to the published HAdV-46 100 kDa protein. Protein VIII is a minor capsid protein that plays a role in the stability of the virion capsid [50]. The pVIII protein of HAdV-37 is 24.6 kDa in molecular weight and has a 99% identity to the published HAdV-46 pVIII protein. The 22 kDa protein is involved in the packaging of HAdV DNA [51]. A 22 kDa homologue was identified in HAdV-37 with a predicted protein of 137 amino acids and molecular weight of 15.8 kDa. Its highest percent identity was to the HAdV-9 22 kDa protein (99%). The predicted polyadenylation signal for the L4 transcription unit is at nucleotide 26507.

The L5 region of the HAdV genome consists entirely of the fiber protein gene. Fiber protein trimerizes to produce the functional unit which projects from the 12 penton vertices of the virus capsid. The fiber protein's carboxyl (C)-terminal globular domain, known as the fiber knob, acts as the primary ligand for host cell receptor binding. The HAdV-37 fiber genome sequence was previously reported, and our predicted protein of 365 amino acids was identical [52]. The HAdV-37 fiber was only 76% identical and 89% similar to its homologue in the HAdV-9 genome (Table 2). The polyadenylation signal for this transcript was predicted at nucleotide 32143. Nucelotide sequence encoding a potential heparan binding site, previously reported in the fiber shaft of HAdV-5, was not present in the HAdV-37 fiber gene [53-55].

### Virus-associated RNA

Most HAdVs contain two virus-associated (VA) RNA genes, VA RNAI and VA RNAII. VA RNAI acts against cellular antiviral defense by blocking the activation of the protein kinase PKR, which when activated turns off protein synthesis in infected cells [56]. VA RNAII binds to RNA helicase A and NF90, the latter a component of the nuclear factor of activated T cells (NFAT) [57]. These VA RNAs also have been recently shown to suppress RNA interference [58]. The VA RNA genes for HAdV-37 were previously identified [59]. Our sequence for VA RNAI is located at nucleotides 10253–10410 and is 99% identical to the previously reported sequence, differing by only one base pair. VA RNAII is located at nucleotides 10471–10620 and was 100% identical to that previously reported.

### Protein and phylogenetic analysis

**Figure 3:**
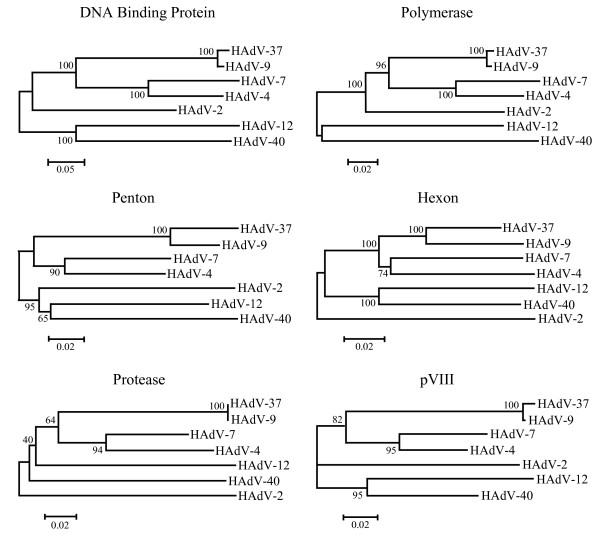
**Phylogenetic analysis of select HAdV proteins.** Bootstrap confirmed neighbor joining trees designed from MEGA 3.1 demonstrate phylogenetic relationships between select proteins of HAdV-37 and representative homologues from each of the 6 HAdV species. The Gonnet protein weight matrix in ClustalX alignment was used, along with complete deletion options. Bootstrap confidence levels (500 replicates) are shown as percentages on the relevant branches.

The annotation of the HAdV-37 genome allows for its comparison with other HAdV serotypes within species D as well as serotypes from other species. Percent identity and similarity of predicted proteins from each of the major transcription units were identified for representative serotypes using Fasta3 [60], and are shown in Table 2. In this analysis, highest identities outside of species D were seen with species B (HAdV-7) and species E (HAdV-4) viruses. Projected protein sequences were then subjected to phylogenetic analysis using Molecular Evolutionary Genetics Analysis (MEGA) 3.1. Bootstrap confirmed neighbor joining trees also suggested that outside of HAdV species D, the serotypes phylogenetically closest to HAdV-37 were within HAdV species B and E (Figure 3). We further selected specific proteins for analysis by virtual 2D gel (JVirGel 2.2.3b) [61,62], based on ClustalW alignments of predicted protein amino acid sequences comparing serotypes from different HAdV species. The accuracy of these virtual 2D gels with regards to pI has been judged to be within ± 1 pI unit of the true migration of the physical protein, even when subsequent post-translational modifications are taken into account [61,63,64].

Migration patterns for select protein homologues in the virtual 2D gel showed projected differences in size and/or pI (See Additional file 1: Supplemental figure 4). The HAdV-37 DNA binding protein migrated to a predicted molecular weight of 54.9 kDa and a pI of 8.52 (Table 3 and Additional file 1). The range of pI for the DNA binding protein among all serotypes tested was from 6.30 to 8.57. The DNA polymerase homologues also revealed substantial differences in predicted size among the selected serotypes, and a range in pI from 6.19 to 8.18 (Table 3). HAdV-37 and HAdV-9 polymerase both migrated to a predicted molecular weight of 125 kDa with pI's of 6.28 and 6.19, respectively. The HAdV-40 homologue had a predicted pI of 8.14. The predicted molecular weights of the penton and hexon proteins differed between serotypes by less than 10 kDa, with a pI range that was probably within the range of accuracy of the software (Table 3 and additional file 1). The L3 protease homologues migrated to almost identical areas on the virtual gel (Additional file 1), consistent with very high percent similarity between HAdV-37 protease and the other homologues (93 to 100%, Table 2). In contrast, despite high percent similarity in the pVIII protein between HAdV-37 and HAdV-4 (94%), the predicted HAdV-37 pVIII migrated to a pI of 8.80, while the HAdV-4 pVIII migrated to a pI of 6.22 (Table 2 and Additional file 1). Further review of the ClustalW alignment for these 2 homologues revealed that despite their high similarity, there were 3 specific amino acid differences in HAdV-37 that when changed to match the residues in HAdV-4, resulted in a pI for HAdV-37 of 5.78 (G46D, Q57E, Q172E, data not shown).

**Table 3:** Molecular weight/pI of select HAdV-37 proteins and their homologs in other HAdV species

Types/Species	DNA binding protein	DNA polymerase	Penton Base	Hexon	Protease	pVIII
HAdV-12/A	55.0/**7.19**	135.0/**7.50**	56.4/6.29	103.0/5.56	23.5/8.50	25.3/9.42
HAdV-7/B	58.2/8.57	136.0/6.87	61.9/5.37	105.7/5.13	23.7/8.69	24.9/**6.12**
HAdV-2/C	59.1/8.06	120.4/6.22	63.2/5.10	109.2/4.81	23.1/8.68	24.7/9.52
HAdV-9/D	54.8/8.52	125.4/6.19	58.6/5.21	106.3/5.00	23.6/8.61	24.6/8.15
HAdV-37/D	54.9/8.52	125.4/6.28	58.4/5.47	106.8/5.6	23.4/8.61	24.6/8.80
HAdV-4/E	57.2/8.11	116.7/6.33	60.0/4.92	105.4/5.25	23.4/8.13	24.7/**6.22**
HAdV-40/F	53.3/**6.30**	135.1/**8.14**	56.9/5.84	104.0/5.74	23.3/8.68	25.3/8.47

Values in bold represents pI's +/- > 1 pH unit from predicted HAdV-37 proteins

Molecular weight and pI were calculated using JVirGel 2.2.3b

### Hypothetical proteins

During annotation of HAdV-37, we located 8 hypothetical ORFs similar to ORFs predicted from sequences previously archived in GenBank for other HAdVs (Table 4), with a blast value for each of less than e^-5^. GeneMark identified one of these putative proteins (HAdV-7 13.6 kDa agnoprotein), and JCVI's annotation engine identified another (E3B 31.6 kDa), while the rest were identified by NCBI's ORF finder. Four of the 8 proteins were located on the complementary strand and 5 were clustered in the area between the intermediate and late ORFs.

**Table 4:** Conserved hypothetical HAdV-37 Proteins

Predicted Size	Blast Result	E-Value	Accession Number
20.5 kDa	HAdV-5 Hypothetical 12 kDa	5.0 E-20	AAW65500
34.76 kDa	HAdV-7 Hypothetical 10.4 kDa	7.0 E-23	AAW33389
16.57 kDa	HAdV-7 13.6 Agnoprotein	2.0 E-39	AAT97609
19.29 kDa	HAdV-16 Hypothetical 12.6 kDa	9.0 E-12	AAW33435
11.4 kDa	HAdV-3 Hypothetical 9.7 kDa	1.0 E-9	AAW33161
8.58 kDa	HAdV-7 Hypothetical 11.3 kDa	7.0 E-14	AAT97539
9.29 kDa	HAdV-7 Hypothetical protein	2.0 E-20	AAT97549
31.6 kDa	HAdV-9 E3 orf3 33.1 kDa	3.0 E-65	CAI05981

Blast results were obtained by blastp using default parameters.

## Discussion

We have determined the complete 35,213 base pair genome of HAdV-37 and identified 35 putative adenoviral genes along with 8 hypothetical ORFs conserved with at least one other HAdV for each ORF. Comparison of the HAdV-37 genome to that of HAdV-9, another species D virus, identified areas of substantial divergence in the penton, hexon, E3, and fiber regions. Disparities between these two HAdV species D viruses in genes encoding structural and host receptor-binding proteins were somewhat expected and also consistent with known differences in host tissue tropism, for example the propensity of HAdV-37 to cause corneal infection, as compared to the association of HAdV-9 with urethritis and follicular conjunctivitis [7,65]. Differences between HAdV-9 and 37 in the E3 region, known to be important to immune evasion and regulation by the virus, but not essential to viral replication *in vitro*, suggest as yet undiscovered functions for this region [22,23]. Divergence in the E3 region, possibly relevant to cellular and tissue specificity during infection, might be due to positive selection. Sequencing of other HAdVs within species D would provide further insight into this area of the HAdV genome.

By phylogenetic analyses and paired comparisons of predicted proteins, HAdV-37 and HAdV-9 of species D appeared most closely related to HAdV-7 of species B and HAdV-4 of species E. Subsequent virtual 2D gel analyses suggested that for a few proteins, a relatively few amino acid substitutions between otherwise similar proteins conferred significant effects on protein charge. If our analyses prove correct, such differences suggest that the function of such proteins in HAdV species D could be quite different than previously described for serotypes of other HAdV species. We acknowledge that our predictions represent a first approximation of protein characteristics, and could be subject to over-interpretation for at least two reasons. First, our comparisons to other viruses are only as reliable as the quality of GenBank viral sequence and annotation. Secondly, post-translational modifications may alter both charge and molecular weight of any given protein. Actual 2D gel analysis will be necessary to confirm such predicted differences.

There is growing concern over the accuracy of *in silico *ORF prediction in AdVs due to splice variants, as well as inconsistencies in banked annotations [66]. To address such concerns, we compared HAdV-37 annotation using three different methods: NCBI ORF finder, JCVI's annotation engine, and GeneMark Heuristic model. We narrowed our annotation to 35 ORFs by comparison with previously determined adenoviral annotations, but we consider our annotation provisional. We identified 8 hypothetical ORFs similar to those previously identified in other HAdV species. The very suggestion of hypothetical proteins implies that our understanding of the HAdV is far from complete. Transcriptome analysis using viral microarrays may help to clarify the best annotation [67]. We suggest that the true transcriptome and proteome of HAdV-37 remain to be determined.

Future sequencing of HAdVs may permit new insights into viral origin, evolution, and pathogenesis. Recently, HAdV-22 was isolated for the first time from an outbreak of epidemic keratoconjunctivitis. The HAdV-22 isolate was shown to contain both HAdV-8 fiber gene and HAdV-37 penton base gene [68]. These recombination events apparently conferred corneal tropism to HAdV-22, a virus not normally known to infect the cornea. As more HAdV species D viruses are sequenced, new insights into tropism and pathogenesis are likely to emerge.

## Conclusion

In summary, the complete genome sequence of HAdV-37 was determined and annotated. The organization of the HAdV-37 genome is similar to other human species D adenoviruses except in the penton, hexon, E3, and fiber regions. Phylogenetic analysis of HAdV-37 proteins revealed close relation to species B and E human adenoviruses, while virtual 2D gel analysis identified differences in proteins thought to function similarly. The availability of the HAdV-37 complete genome sequence will facilitate future studies into the pathogenicity of this important human pathogen.

## Methods

### Cells, virus stock, DNA purification

HAdV-37 strain GW was obtained from the American Type Culture Collection (ATCC). Virus stocks were grown in A-549 cells (CCL-185), a human alveolar epithelial cell line that was previously shown to support HAdV-1 virion production [69]. Virus was purified by CsCl gradient and subsequent dialysis, and stored at -80°C. DNA extraction was accomplished by the addition of proteinase K, phenol:chloroform extraction, and finally ethanol precipitation.

### Sequencing

Standard PCR methodology was used to amplify regions of the genome to be sequenced. HAdV type 17 was used as a reference strain for the design of initial PCR primers. To close gaps in the sequence and improve overall sequence quality, Primer 3 [70] and CONSED [71] software were used to design primers from newly acquired sequence. Shrimp alkaline phosphatase and exonuclease I treatment were used to dephosphorylate and degrade residual PCR primers present together with the PCR products. Sequencing was performed using the ABI BigDye Terminator v3.1 cycle sequencing kit (Applied Biosystems, Foster City, CA). The sequencing reaction mixture was purified using Sephadex G-50 (Sigma Aldrich, St. Louis, MO), and the reaction products analyzed on ABI 3700 or ABI 3730 XL capillary electrophoresis DNA sequencers (Applied Biosystems). To sequence the viral inverted terminal repeat (ITR) ends, primers were designed from newly determined adjacent sequence, and direct sequencing was performed using whole genome DNA as the template [69].

### Sequence analysis and genome annotation

Sequence data was filtered using LUCY (JCVI, Rockville, MD), and data assembly performed with Phred/Phrap, using default assembly parameters [71-73]. Genome assembly contained 664 high quality reads with an average length of 834 bps. The fold coverage for both strands of the genome was 15. The Phrap average quality score was 89.0. Genome annotation was performed using JCVI's automated annotation system [74], and the data was stored in a MySQL database. Manatee [75] was used to manually review the data from the annotation engine. Additionally, we used GeneMark Heuristic Models gene prediction [76], and NCBI's ORF Finder [77] to examine the sequence. Open reading frames were searched against available databases in GenBank, PIR, SWISS-PROT, and JCVI's CMR database. Splice sites were predicted using a splice site finder program [34]. An online sequence alignment program, mVISTA LAGAN [78] was used for global pair-wise sequence alignment [15]. CpG analysis was performed with FUZZNUC [12].

### Nucleotide sequence accession numbers

The nucleotide sequence for the following HAdVs can be found in GenBank: HAdV-2 [AC_000007], HAdV-4 [AY487947], HAdV-7 [AC_000018], HAdV-9 [AJ854486], HAdV-12 [AC_000005], HAdV-17 [AC_000006], HAdV-40 [L19443]. Previously sequenced HAdV-37 penton base protein, hexon protein, fiber protein, and VA RNA gene accession numbers are AAG00906, ABA00016, AAB71734, and U10679, respectively. The GenBank accession number for HAdV-37 is DQ900900.

### *In silico *protein analysis

Percent identities and similarities between proteins of HAdV-37 and other HAdVs were determined using Fasta3 [60,79] and Blastp software [80]. Proteins from the GenBank database were analyzed by an *in silico *2D gel program JVirGel 2.2.3b [61]. Phylogenetic analysis was performed with Molecular Evolutionary Genetics Analysis (MEGA) 3.1 [81]. Bootstrap confirmed neighbor joining phylogenetic trees were designed with MEGA 3.1 with 500 replicates.

## Authors' contributions

CMR designed primers, annotated the virus, performed the bioinformatics analysis, and drafted the manuscript. FS performed the PCR, and assisted with compilation of the sequence. AFG and DWD participated in primer design, sequence compilation and analysis, and manuscript writing. JC conceived the project design, and participated in the data analysis writing of the manuscript. All authors read and approved the final manuscript.

## Supplementary Material

Additional file 1 2D Gel Analysis. Virtual 2D gel analysis. Protein migration patterns for select HAdV proteins by virtual 2D gel. Each spot represents a given serotype's homologue based on its predicted amino acid sequence. A. DNA binding protein, B. Viral polymerase, C. Penton base, D. Hexon, E. Protease, and F. pVIII. One protein from HAdV-37 and a homologue from a representative serotype of all 6 HAdV species are represented in each gel. Click here for file
