# Supplementary material for: Genomic and bioinformatics analysis of human adenovirus type 37: New insights into corneal tropism
Source: BMC Genomics. 2008 May 9;9:213. doi: 10.1186/1471-2164-9-213 (PMC2397415; doi:10.1186/1471-2164-9-213)
Supplement: Additional file 1 — 2D Gel Analysis. Virtual 2D gel analysis. Protein migration patterns for select HAdV proteins by virtual 2D gel. Each spot represents a given serotype's homologue based on its predicted amino acid sequence. A. DNA binding protein, B. Viral polymerase, C. Penton base, D. Hexon, E. Protease, and F. pVIII. One protein from HAdV-37 and a homologue from a representative serotype of all 6 HAdV species are represented in each gel. [file 1471-2164-9-213-S1.ppt]

## Slide 1
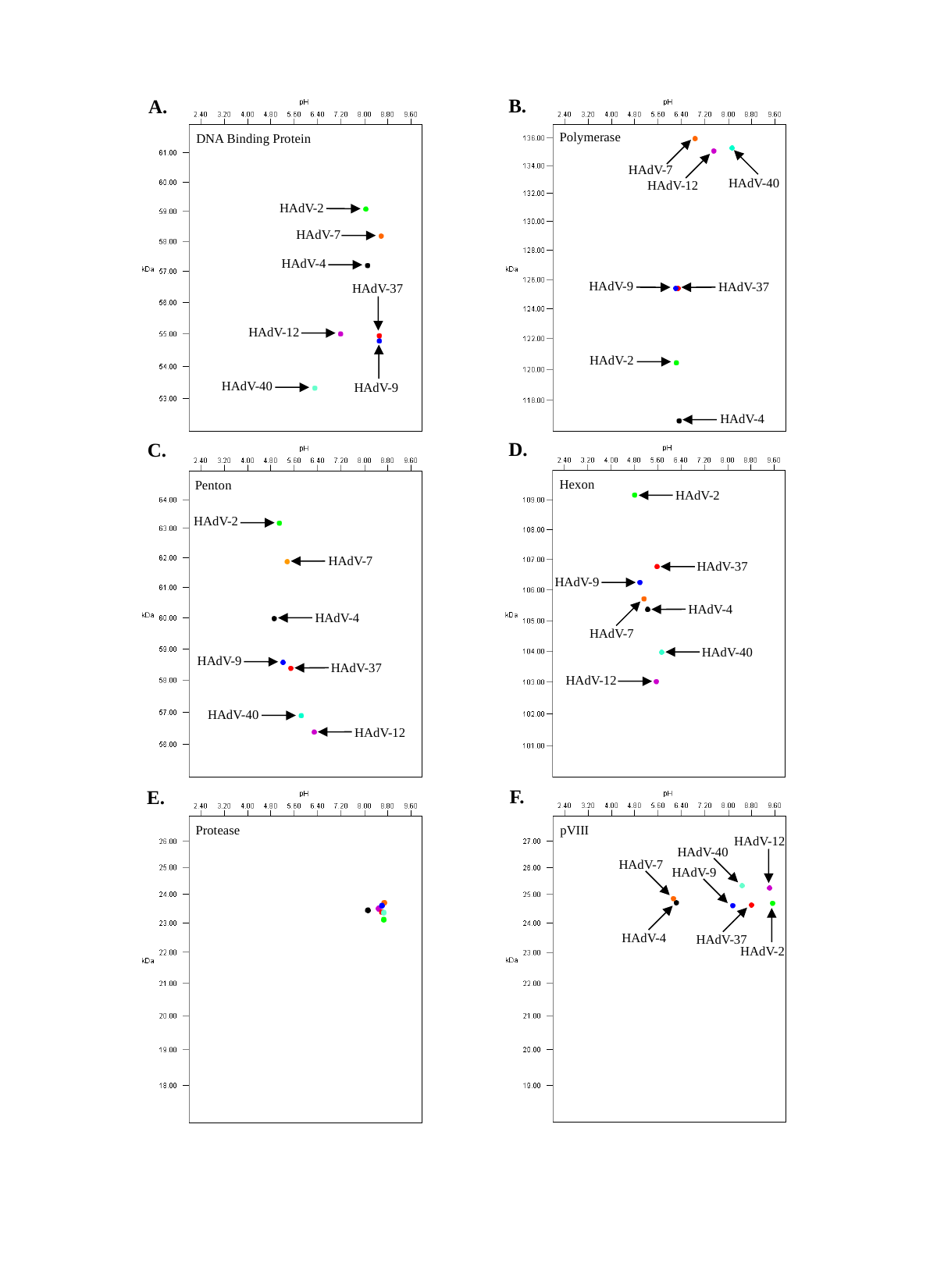

B.
A.
Polymerase
DNA Binding Protein
HAdV-7
HAdV-40
HAdV-12
HAdV-2
HAdV-7
HAdV-4
HAdV-9
HAdV-37
HAdV-37
HAdV-12
HAdV-2
HAdV-40
HAdV-9
HAdV-4
D.
C.
Hexon
Penton
HAdV-2
HAdV-2
HAdV-7
HAdV-37
HAdV-9
HAdV-4
HAdV-4
HAdV-7
HAdV-40
HAdV-9
HAdV-37
HAdV-12
HAdV-40
HAdV-12
F.
E.
Protease
pVIII
HAdV-12
HAdV-40
HAdV-7
HAdV-9
HAdV-4
HAdV-37
HAdV-2
